# Dataset of damaged photovoltaic cells electroluminescence, visible and near-infrared multispectral images with performance degradation data and LLM-RAG descriptors

**DOI:** 10.1016/j.dib.2026.113109

**Published:** 2026-07-27

**Authors:** Marcello Polenghi, Vittorio Sala, Emanuele Ogliari, Roberto Caldelli, Claudio Loconsole, Anna Valente, Ambra Vandone

**Affiliations:** a Università Telematica Universitas Mercatorum Piazza Mattei, 10-00186 Roma, Italy; b Scuola universitaria professionale della Svizzera italiana (SUPSI) - Dipartimento tecnologie innovative - Polo universitario Lugano - Campus Est USI-SUPSI, Via la Santa 1, Lugano-Viganello, CH, 6962, Switzerland; c Politecnico di Milano, Dipartimento di Energia, Via Raffaele Lambruschini, 8, 20156, Milan, Italy; d CNIT, Viale Morgagni, 65, 50134, Florence, Italy; e Scuola Superiore Sant’Anna, Piazza Martiri della Libertà, 33, 56127, Pisa, Italy

**Keywords:** PV solar modules, EL, Large language models, Retrieval augmented data, HDR, Image database, Predictive maintenance, LLM-RAG

## Abstract

This dataset provides comparative images and power output performance records of damaged photovoltaic (PV) cells, along with an extensive textual description of each degradation and power loss level. The dataset is limited to a small number of cells from a single photovoltaic technology, as it is designed for controlled multimodal correspondence rather than statistical generalization. Each PV cell (DAUERHAFT Polycrystalline AP130 × 150 MM 4.5 [Wp]), originally brand-new, was deliberately subjected to progressive damage caused by soiling and by mechanical actions such as direct impact from various blunt objects, cuts, abrasions, scrapes, bending, smashing, cracking, as well as thermally and electrically induced degradation, including direct exposure to flames and Joule effect overheating resulting in a fire. Under each condition, a set of three images was acquired, using different techniques: visible high-dynamic-range (HDR) under artificial uniform light conditions; near-infrared monochrome (NIR) under the same light conditions; electroluminescence (EL) without any external light source, but connecting the PV cell to a direct current power source current controlled.

During EL, the imposed current and resulting voltage were recorded directly by the power source system. Then, after unplugging the power source, short-circuit current and open-circuit voltage were acquired under a standard test light source.

Data was acquired in-door, using a laboratory electric power source, a digital multimeter, an HDR camera (for visible-HDR images), and a filtered visible/near-infrared camera (BP800 filter, for both NIR and EL images). Cameras were fixed in a binocular setup and optically calibrated with a calibration board. Using the same camera for NIR and EL resulted in an almost perfect pixel-pixel correspondence of images.

The dataset highlights a significant difference between the visual information obtained by various imaging technology, showing the relevance of a multi-spectral approach as well as the valuable insight in degradation evaluation given by the EL when electrical parameters are recorded simultaneously.

The dataset combines pixel-level aligned HDR, monochrome, and electroluminescence images with electrical measurements and natural-language descriptions of degradation conditions. Existing photovoltaic datasets do not appear to provide this specific combination of aligned imaging modalities, electrical data, and defect annotations. The dataset may support research on cross-modal correspondence learning and multimodal approaches that jointly exploit visual, electrical, and linguistic information.

©2026 The Authors. Published by Elsevier Inc. This is an open access article under the CC BY license (http://creativecommons.org/licenses/by/4.0/)

Specifications Table

**Table:** 

Subject	Engineering & Materials science
Specific subject area	Indoor undistorted multispectral imaging and testing of PV cells under incremental fault conditions with power output records and textual annotations.
Type of data	Raw data: Table (.csv) Processed data: Table (.csv), Image (.png) Analyzed data: Table (.csv)
Data collection	Data were acquired experimentally in SUPSI-DTI-ARM lab over a time span of 60 days since November 2025. Two different PV cells were selected to acquire multiple images and electrical information under various damage conditions (soiling, mechanical, and thermal). **Testing Material**: i) Two PV cells AP130 × 150 MM 4.5W ii) KORAD KD3005D DC power supply iii) OSRAM DECOSTAR 51 ALU G5.3 20 W 12 V 36° halogen lamp **Cameras**: optically calibrated binocular setup. i) LUCID Triton HDR5.4, ii) IDS u3–36p0xcp with MidOpt BP800 bandpass filter **Measuring Instruments**: Metex Corporation M-3660D multimeter **Software**: MVTec HALCON Campus for automatic cropping and alignment
Data source location	**Institution**: Scuola universitaria professionale della Svizzera italiana (SUPSI) - Dipartimento tecnologie innovative - Polo universitario Lugano - Campus Est USI-SUPSI - Via la Santa 1, Lugano-Viganello CH-6962.
Data accessibility	Repository name: PV_EL_HDR_BW_Test_DB Data identification number: 10.17632/pxyzzkbs4b.1 Direct URL to data: https://data.mendeley.com/datasets/pxyzzkbs4b/1
Related research article	none

## 1 Value of the Data

• This dataset offers a direct comparison between multiple images of the same PV cell taken with different techniques and the corresponding electrical parameters in different damage conditions, as well as the relative textual descriptors. It gives valuable insights into PV cells' performances in real-world fault conditions, including soiling, mechanical, thermal, and fire damage.
• The dataset includes PV cells' electroluminescence (EL) images, visible light HDR images, near-IR monochrome images, and detailed electrical data such as voltage, current, and power output in different damage conditions.
• The dataset includes categorical filters and extensive text descriptions of each damage condition, how it appears on the image, and the corresponding output power loss
• The integration of pixel-level aligned multispectral images, electrical measurements, and natural-language descriptions provides a multimodal dataset for the study of photovoltaic (PV) cell characteristics from complementary sensing modalities.
• The dataset may support investigations of relationships between imaging data, electrical response, and defect descriptions, as well as research on image analysis, computer vision, and multimodal learning methods. In particular, it enables studies on correspondence between electroluminescence (EL) and visible-spectrum imaging, including approaches for generating EL-like representations from non-EL images.
• A key aspect of the dataset is the availability of precisely aligned multimodal observations under controlled degradation conditions, which may facilitate research on cross-modal relationships between imaging modalities and reduce reliance on repeated electroluminescence acquisitions in laboratory environments, which are generally associated with dedicated laboratory setups and non-negligible experimental cost.

## 2 Background

Photovoltaic (PV) systems performance analysis [1] is important for techno-economic evaluations. Monitoring PV modules maintenance level, degradation, and performance decay [2] allows for deeper insight into the PV plant productivity, especially in the context of comparative analyses among different PV applications [3], when forecasting power output [4], multi-user configuration [5,6], or complex system installations [7].

Aerial imaging of PV plants [8] has recently gained importance for module defects detection [9], O&M [10], and automatic monitoring [11], simplifying data collection and allowing remote analysis.

Laboratory-based electroluminescence (EL) testing is considered the most informative diagnostic technique; however, it cannot be readily applied in on-field conditions. Several benchmark image datasets [12] support EL image machine learning and deep learning processing, including segmentation [13], defect classification [14], fault detection [15], and synthetic images generation [16].

However, existing EL reference datasets lack a systematic association between visible-light and corresponding EL images, as well as associated electrical parameters and textual descriptions. Therefore, applicability to real-world PV plants is limited.

Leveraging previous experiences in image acquisition in PV context [17,18] and with multispectral cameras [19], a new dataset [20] has been produced to fill this gap.

Recent contributions have explored complementary aspects of photovoltaic module diagnostics, degradation characterization, and electroluminescence imaging datasets [[21], [22], [23]]. Together, these studies highlight the diversity of available data resources and methodologies within the photovoltaic research community, further demonstrating the growing interest in data-driven approaches for PV analysis.

## 3 Data Description

### 3.1 Overview

This dataset contains high-resolution (500 DPI) images and experimental measurements of photovoltaic cells' power output degradation under progressively induced different damage conditions. A limited set of 2 PV cells was subjected to a random sequence of deliberate damage actions of various types (mechanical, thermal, electric and dirt), until the complete failure of each PV cell. Due to the random damage action and sequence, complete failure of each PV cell took place after an a-priori unpredictable number of physical damage or detrimental effects. This procedure lead to a total of 84 different damage conditions, unevenly distributed across the cells. The sequence, type, and effects of the damage actions are reported and described in detail in the data files included in the database, as described in the following tables. The damage sequence was deliberately chosen with a random approach and variable typology in order to represent a real-world condition and eliminate possible biases due to the specific damage sequence eventually selected. In order to increase variability and resemblance to reality, some damage conditions were randomly corrected by removing a variable amount of soot, dust, or residue from previous damage actions, simulating the effect of wind, rain, or manual cleaning. This condition of cumulative or non-cumulative damage is reported in the DB data files as well. Each damage condition is described by a 3-images-set (Near-IR Electroluminescence, monochrome Near-IR, Visible HDR for a total of 252 images); for each damage level are also reported the corresponding performance measurements under controlled conditions and a detailed verbal description, encompassing both categorical and qualitative aspects.

The dataset includes:

- 84 Experimental 3-image-sets (total 252 images; each image-set is composed by 3 images: HDR, EL, BW) (PNG format)

- Image files data (CSV)

- Electrical performance measurements (voltage, current, power output) and verbal description of damage as identified through visual inspection (CSV), damaging sequence, damaging procedure and damage type and effect

- Metadata describing cell type, naming conventions, experimental parameters (TXT)

### 3.2 Dataset structure and data description

**Fig. 1:**
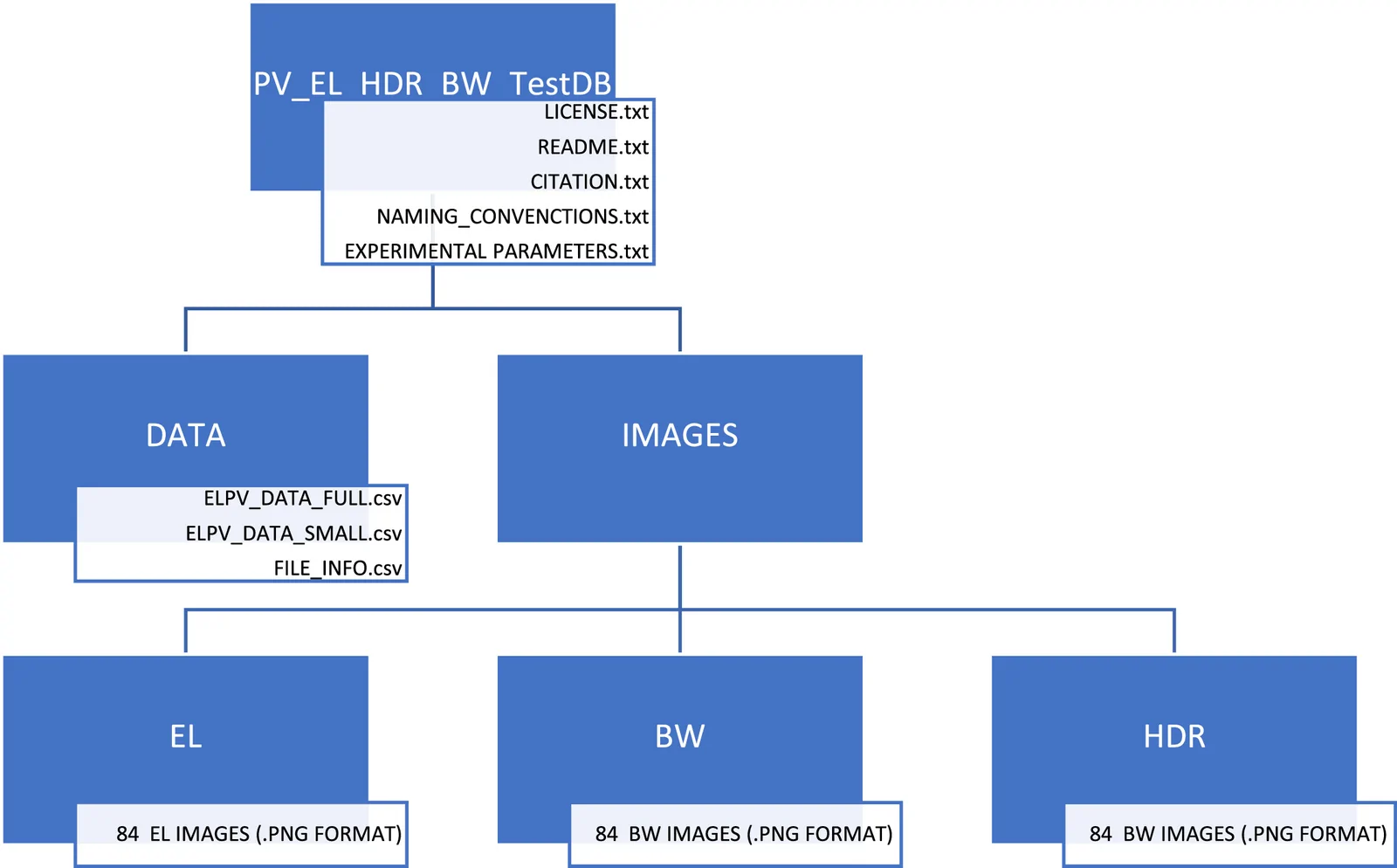
Distribution of folders in the database.

The dataset structure is organized as shown in Fig. 1.

where:

“PV_EL_HDR_BW_TestDB” is the main root folder and contains supporting .txt documents

• LICENSE.txt (legal claims)
• README.txt (a summary of all relevant information about the dataset)
• CITATION.txt (suggested citation format, authors' info, and keywords)
• NAMING_CONVENCTIONS.txt (specific details on .csv file’s structure and definitions)
• EXPERIMENTAL PARAMETERS.txt (specific details of experimental parameters)
• 2 subfolders: “data” and “images”

“data” subfolder contains .csv data files

• ELPV_DATA_FULL.csv (Full Dataset with all numeric data and extensive textual damage description for LLM-RAG)
• ELPV_DATA_SMALL.csv (A condensed dataset version including solely numerical data and the essential textual annotations describing damage)
• FILE_INFO.csv (Image files metadata table)

“images” subfolder contains 3 subfolders that respectively include:

• EL/ # 84 EL images (PNG): Electroluminescence Near-Infrared images (obtained with a Visible light calibrated camera with BP800 filter)
• BW/ # 84 BW images (PNG): Monochrome Near-Infrared images (obtained with a Visible light calibrated camera with BP800 filter)
• HDR/ # 84 HDR images (PNG): High Dynamic Range images (obtained with a Visible HDR calibrated camera)

**Fig. 2:**
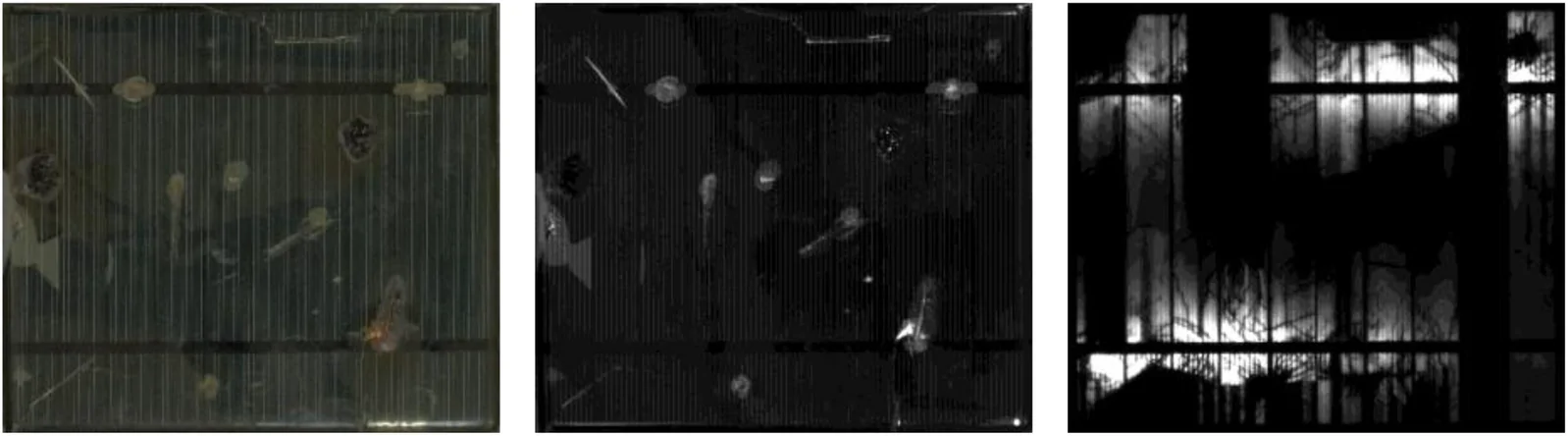
Example 3-image-set of a damaged PV cell: HDR, monochrome NIR, EL.

Thus, each cell in each damage condition is described with a set of 3 images (Fig. 2) with perfectly corresponding pixel dimensions (slightly variable among the 3-image-sets due to fine cropping and alignment). No resizing has been applied. Between monochrome NIR and EL there is also a perfect pixel-to-pixel alignment since they have been acquired with the same camera at the same time.

### 3.3 Files and folders naming rules

Referring to the data structure and files list presented, naming rules are reported in the following (Table 1, Table 2, Table 3).

**Table 1:** images/ folders naming rules.

Directory	Sudirectory	Description	Details
images/	EL	Electroluminescence Near-Infrared PNG images	obtained with a Visible light camera with a BP800 filter
images/	BW	Monochrome Near-Infrared PNG images	obtained with a Visible light camera with a BP800 filter
images/	HDR	High Dynamic Range color images	obtained with a Visible HDR camera

**Table 2:** images/ subfolders (EL,BW,HDR) files naming rules.

	<CellID>_<DamageID>_<ImageTypeID>_rt_cr.png
<CellID>	Unique identifier for each panel (may be "0.1″ or "0.2")
<DamageID>	May be S# for soiling and D## for all other damage types (# one digit number; ## two-digit number)
<ImageTypeID>	May be "EL" for Electroluminescence, "BN" for NIR and "HDR" for High Dynamic Range
_rt_cr	Indicates that the images have been rectified and cropped to get the best pixel-pixel correspondence (perfect between NIR and EL - same camera)

**Table 3:** Data/ folder files naming rules.

	ELPV_DATA_<SIZE>.csv
<SIZE>	may be "FULL" for the complete dataset or "SMALL" for the condensed dataset

Metadata file FILE_INFO.csv includes image-file-specific metadata (file name, image type (EL,BW,HDR), file type, file size [KB], image width and height [pixels], image DPI, image bit size)

### 3.4 CSV data files variables naming

Referring to the CSV data files list presented, each damage condtion is described by a set of features. Features, variables descriptons and textual descriptors details are reported in the following (Table 4, Table 5, Table 6, Table 7):

**Table 4:** CSV data files variable naming (ELPV_DATA_FULL.csv and ELPV_DATA_SMALL.csv).

Variable	Units / Format	Description
images_set_code	7digit-text-code	Unique identifier for the file
images_set_name	6to7digit-text-code	Unique name of 3-image set
EL_file_name	text	Unique identifier for the electroluminescence file of the 3-image set
PV_cell_name	3digit-text-code	Unique identifier for the photovoltaic cell
EL_file_Width [px]	pixels	Electroluminescence image width
EL_file_Height [px]	pixels	Electroluminescence image height
V_EL [V]	Volts (V)	Input voltage applied during the electroluminescence
A_EL [mA]	MilliAmperes (mA)	Input current measured during the experiment
V_alo_light [V]	Volts (V)	Output voltage measured during operation under a halogen test lamp
A_alo_light [mA]	MilliAmperes (mA)	Output current measured during operation under a halogen test lamp
P_alo_light	MilliWatts (mW)	Computed output power measured during operation under a halogen test lamp
Power_output_loss [%] [V*I]	%	Computed output power percentage loss measured during operation under a halogen test lamp
Degradation_level	Categorical	Qualitative degradation level (see DAMAGE LEVELS table)
incremental_damage_kind	Categorical	Qualitative text description of incremental damage
incremental_damage_HDR_visible_type⁎⁎⁎	Text	Qualitative text description of incremental damage visible in HDR image of the 3-image set
incremental_damage_BN_visible_type⁎⁎⁎	Text	Qualitative text description of incremental damage visible in BW image of the 3-image set
incremental_damage_EL_visible_type⁎⁎⁎	Text	Qualitative text description of incremental damage visible in EL image of the 3-image set
incremental_damage_cause⁎⁎⁎	Text	Qualitative text description of damage cause
progressive_damage	boolean	Boolean classification: 1 if the damage visible in EL can be used to cumulatively calculate the total damage; 0 if not (i.e. previous damages like soot have been removed)
superposed_incremental_EL_damage	boolean	Boolean classification: 1 if the damage visible in EL is superposed to previous ones; 0 if not
New_Damage_Xmin	pixel	Incremental damage bounding box top left corner X coordinate
New_Damage_Ymin	pixel	Incremental damage bounding box top left corner Y coordinate
New_Damage_Xmax	pixel	Incremental damage bounding box bottom right corner X coordinate
New_Damage_Ymax	pixel	Incremental damage bounding box bottom right corner Y coordinate
New_Damage_position_bounding_box	[Xmin,Xmax,Ymin,Ymax]	Incremental damage bounding box
incremental_damage_description_details⁎⁎⁎	Text	Extensive qualitative text description of incremental damage and text comparison between the images with qualitative assessment of degradation type and causes

⁎⁎⁎ indicates a field not present in ELPV_DATA_SMALL.CSV. **Missing values: `NaN`.

**Table 5:** Damage type synthetic descriptors.

DAMAGE LEVELS	POWER OUTPUT [V*I] LOSS [%]
NONE	0%
LIGHT	<5%
MINOR	5%−14%
MODERATE	15%−24%
SEVERE	25–44%
CRITICAL	45%>
CATASTROPHIC	100%

**Table 6:** Soiling levels synthetic descriptors.

SOILING LEVELS	POWER OUTPUT LOSS [%]
LOW	0%−5%
MEDIUM	5%−9%
HIGH	10%−19%
VERY HIGH	20%>

**Table 7:** Damage type description keywords.

KEYWORD	DESCRIPTION
Dent / Denting:	Localized indentation caused by mechanical impact that deforms the surface without breaking it.
Surface Abrasion / Scratch:	Wearing away or linear marks on the surface that are caused by friction or scraping.
Coating Delamination:	Partial or full separation of the coating from the substrate reduces protection.
Coating Burning/Melting:	Thermal damage that causes softening or liquefaction of the coating due to excessive heat or combustion.
Soot Deposit / Buildup	Accumulation of fine carbon particles that reduces optical clarity and potentially causes corrosion.
Superficial Coating Scratch	Light scratch confined to the outer coating layer without substrate damage.
Deep Coating Scratch	Scratch penetrating the coating and potentially exposing the substrate beneath.
Coating Abrasion	Progressive removal or thinning of the coating due to repeated mechanical action.
Cut / Laceration	Sharp incision or tear penetrating the coating and substrate, compromising structural integrity.
Cracking (Micro/Macro)	Formation of fine or larger fractures within the coating or the substrate caused by stress or fatigue.
Discoloration / Yellowing	Color or transparency changes due to UV exposure or heat, reducing optical performance.
Corrosion / Oxidation	Chemical degradation of metals, causing rust or pitting.
Multilayer Delamination	The separation between multiple layers of panel materials weakening structural integrity.
Edge Chipping / Breakage	Small chips or fragments broken off edges, often from mechanical impact or handling.
Impact Damage / Shattering	Damage ranging from dents to broken glass caused by mechanical impacts like hail or debris.
Smashing damage	Surface fragmentation, which is characterized by the crumbling or the disintegration of the surface
Bending damage	Permanent deformation of the panel caused by mechanical flexure, resulting in warping or curvature of the surface
Moisture Ingress	Penetration of water into internal layers, causing corrosion and electrical faults.
Biological Fouling	Growth of algae, moss, or lichen reducing light transmission and trapping moisture.
Thermal Fatigue	Damage from repeated heating/cooling cycles causing cracking, warping, or delamination.
Soiling / Dust Accumulation	Deposition of dust and particles that block sunlight and reduce panel efficiency.
Chemical Attack / Etching	Surface degradation caused by exposure to pollutants or harsh chemicals.

## 4 Experimental Design, Materials and Methods

**Fig. 3:**
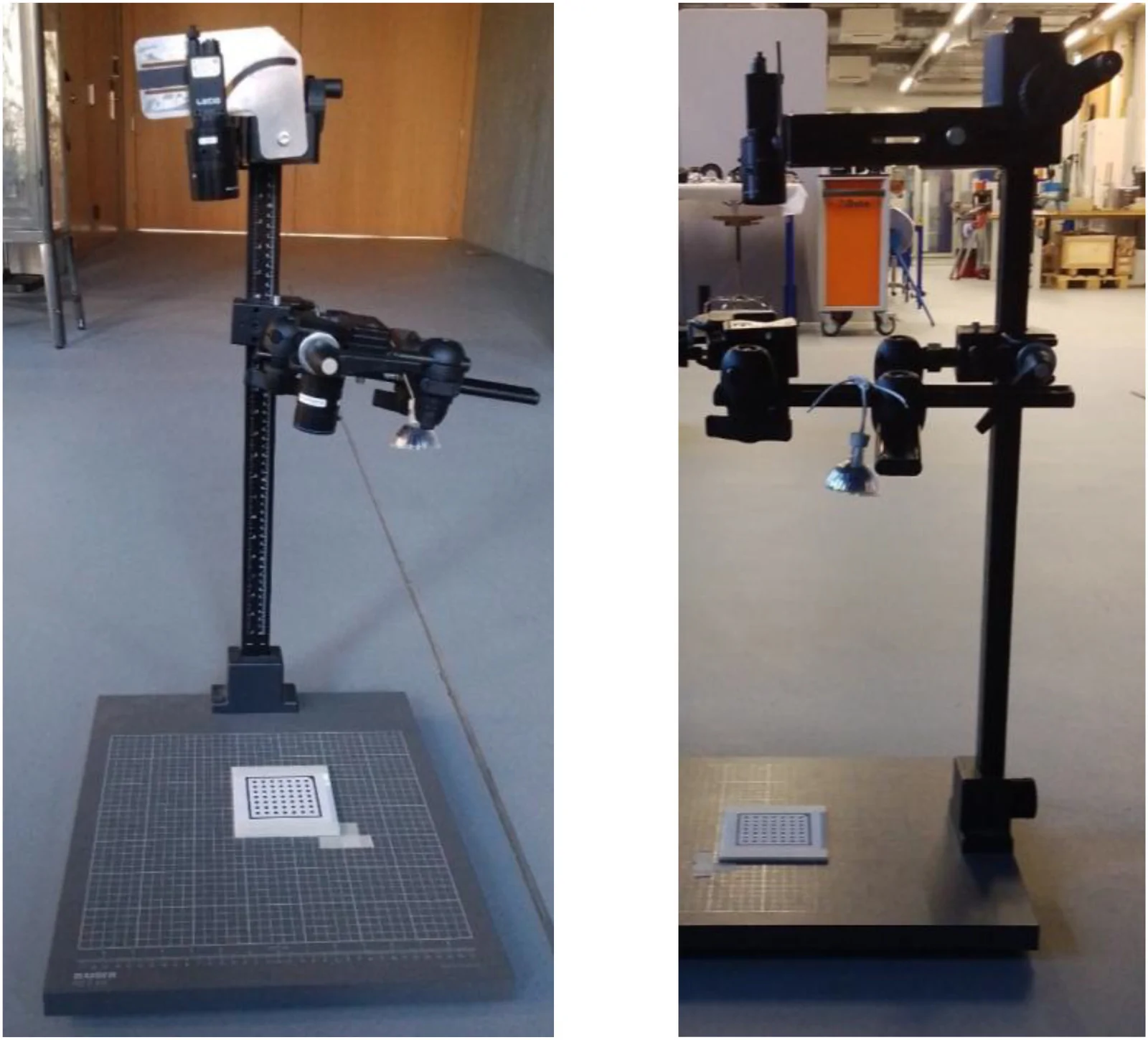
Experimental setup: Y-Z view (left); X-Z view (right);.

Experimental design parameters, materials and methods are described in the following tables (Table 8, Table 9, Table 10, Table 11, Table 12, Fig. 3 and Table 13).

### 4.1 Solar cells specifications

**Table 8:** PV cells specifications.

Parameter	Description	Units / Notes
CellID	Unique identifier	0.1, 0.2
Type	Panel technology	Polycrystalline
Nominal Power Rated power		4.5 Wp (Watts peak)
Nominal Voltage		5V
Nominal Current		0.9A
Dimensions	Physical size	130 mm × 150mm
Manufacturer	Panel manufacturer	DAUERHAFT
Model	PV cell model code	AP130 × 150MM

### 4.2 Measurement protocols overview

**Table 9:** Measurement protocols.

Parameter	Description	Units / Notes
Measurement Interval	Frequency of measurements	5 min between each of the 3-image set
Duration	Experiment duration	80 h; 60-day timespan
Environmental Conditions Monitoring	Ambient temperature/humidity	domestic thermometer/web data
Power Calculation	P = V × I	Watts
Electric Damage Detection	EL imaging of cells	images in `images/EL` visual assessment
Physical Damage Detection	HDR imaging of cells	images in `images/HDR` visual assessment
Soiling Damage Detection	NIR imaging of cells	images in `images/BW` visual assessment
Defect Inspection	Microcracks, delamination etc.	Categorical, visual inspection

### 4.3 Environmental conditions

**Table 10:** Environmental conditions.

Parameter	Range / Notes
Ambient Temperature	12 – 20 °C
Relative Humidity	50 – 70%
Test Lamp Irradiance Power (estimated)	50 W/m²

*Irradiance estimation procedure:*

*Irradiance estimation for a halogen reflector lamp*

*A. Lamp parameters*

*Lamp: OSRAM DECOSTAR 51 ECO SUPERSTAR 47,860 ECO SST 36°*

*Main photometric characteristics:*

• Nominal power: 25 W
• Color temperature: 2900 K
• Color rendering index: Ra = 100
• Beam angle: 36°
• Peak luminous intensity: I₀ = 1000 cd
• Working distance: r = 0.40 m

*The manufacturer reports a peak luminous intensity of 1000* *cd for the 36° reflector lamp.*

*B. Angular geometry*

*The target point is displaced relative to the lamp axis by: X* *=* *10°, Y* *=* *15°; the effective off-axis angle is obtained from direction cosines:* cos*(θ)=* cos*(X)⋅*cos*(Y)=0.951; θ =arccos(0.951) ≈ 18°*

*C. Illuminance at the working distance [cd->lux]*

*Assuming a point-source approximation: Ev=I* cos*(θ)/r^2^, where I = luminous intensity (cd) and* r = distance (m). *Thus E_v_ ≈ 5.9* *×* *103 lx*

*D. Conversion from illuminance (visual, photometric) to irradiance (energetic, radiometric)*

*A commonly used approximation for tungsten-halogen lamps is:* 1W/m^2^≈120 lx

*Thus E_e_ ≈ 50* *W/m^2^*, noting that this value is obtained through a standard photometric-to-radiometric approximation rather than a calibrated irradiance measurement, while constant illumination conditions ensure internal consistency and relative comparability across acquisitionswhich does not affect the intended use of the dataset for multimodal correspondence analysis, which relies on consistent acquisition conditions rather than absolute radiometric calibration.

### 4.4 Equipment - experimental setup

**Table 11:** Equipment - experimental setup.

Device	Model / Notes
HDR Camera	LUCID Triton HDR5.4
Visible/NearIR Camera	IDS u3–36p0xcp
BP800 Filter	MIDOPT BP 800–25.4 (FilBP800/C-mount #24,495)
Test Alogen Lamp	OSRAM DECOSTAR 51 ECO SUPERSTAR 47,860 ECO SST 36°-cone Ra=100 1000 cd 12 V 25 W 2900 K; working distance 40 cm; angles: X = 10°, Y = 15°
Power Supply	KORAD KD3005D DIGITAL-CONTROL DC POWER SUPPLY 0–30 V 0–5A
MultiMeter	Metex Corporation M-3660D digital multimeter

**Table 12:** Camera details.

Image Type	HDR	EL - BW
Camera Type	Visible HDR Camera	Visible/NearIR Camera
Camera Manufacturer	LUCID Vision Labs	IDS Imaging
Camera Model	Triton TDR054S HDR AltaView	U3–36P0XCP-M-GL
Bin	1 × 1	2 × 2
Exposure	80 [ms]	1 [s]
Gain	5 analog	5 analog
Acquisition Software	ArenaView	IDSpeak
Lens	RICOH FL-BC2518–9 M (F5.6/25mm/1.8″)	OPT-CDP-1628-V10 (F2.8/16mm/1.1″/C)
Filter	None	MIDOPT BP 800–25.4 (FilBP800/C-mount #24,495)
Work distance	62 cm (manually measured)	41.5 cm (manually measured)
Angles	X = 5°; Y = 1° (estimated geometrically from setup orthogonal imagery)	X=−7°; Y = 2° (estimated geometrically from setup orthogonal imagery)
CALIBRATION	CALIBRATION BOARD and HALCON software	CALIBRATION BOARD and HALCON software

### 4.5 Calibration and accuracy

Camera calibration by calibration board and Halcon software procedure

**Table 13:** Power supply and sensors accuracy.

Power Supply Voltage accuracy	±0.5% + 20 mV
Power Supply Current accuracy	±0.5% + 10 mA
Multimeter Voltage accuracy	±0.3% of reading + 1 digit
Multimeter Current accuracy	±0.5% of reading + 1 digit
Temperature sensors	unknown °C

### 4.6 Power input values for electroluminescence

V = 6.8 [V]

A= variable 0.7 - 0.9 [A]

Although the nominal datasheet specifies 5 V / 0.9 A, the voltage was increased to 6.8 V to make the electroluminescence clearly visible. The current remained naturally below the nominal maximum because of the device’s nonlinear I–V characteristic: at this voltage, the test cells emitted light efficiently, allowing for limiting image white saturation, without exceeding the nominal current.

### 4.7 Methodology

Overview (Fig. 4):

**Fig. 4:**
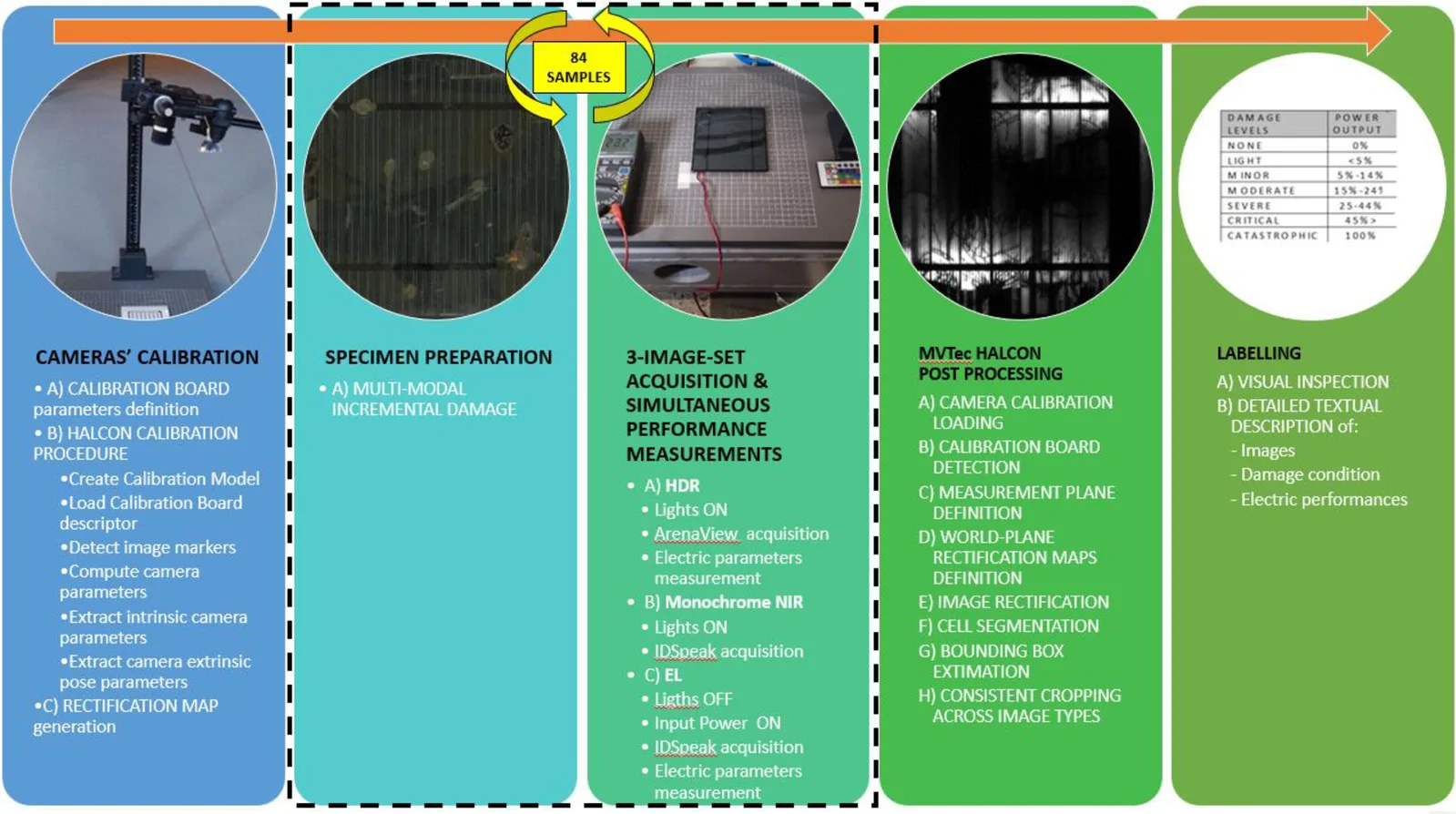
Workflow overview.

Dataset images acquisition procedure is composed by several steps:

1. Cameras’ calibration;
2. Specimen preparation with progressive damage;
3. 3-image set acquisition (HDR, NIR, EL) for each specimen and simultaneous electrical performance measurements;
4. Image post-processing (cropping and alignment);
5. Image labelling with categorical and textual parameters.

1. Cameras’ calibration

The calibration was performed using a calibration board sized 80 × 80 mm, with a grid of equispaced 7 × 7 circular markers, sized 5 mm. An MVTec HALCON .descr file was generated, describing the calibration board geometry (layout type: marker grid, marker size, dots number and spacing between dots).

**Fig. 5:**
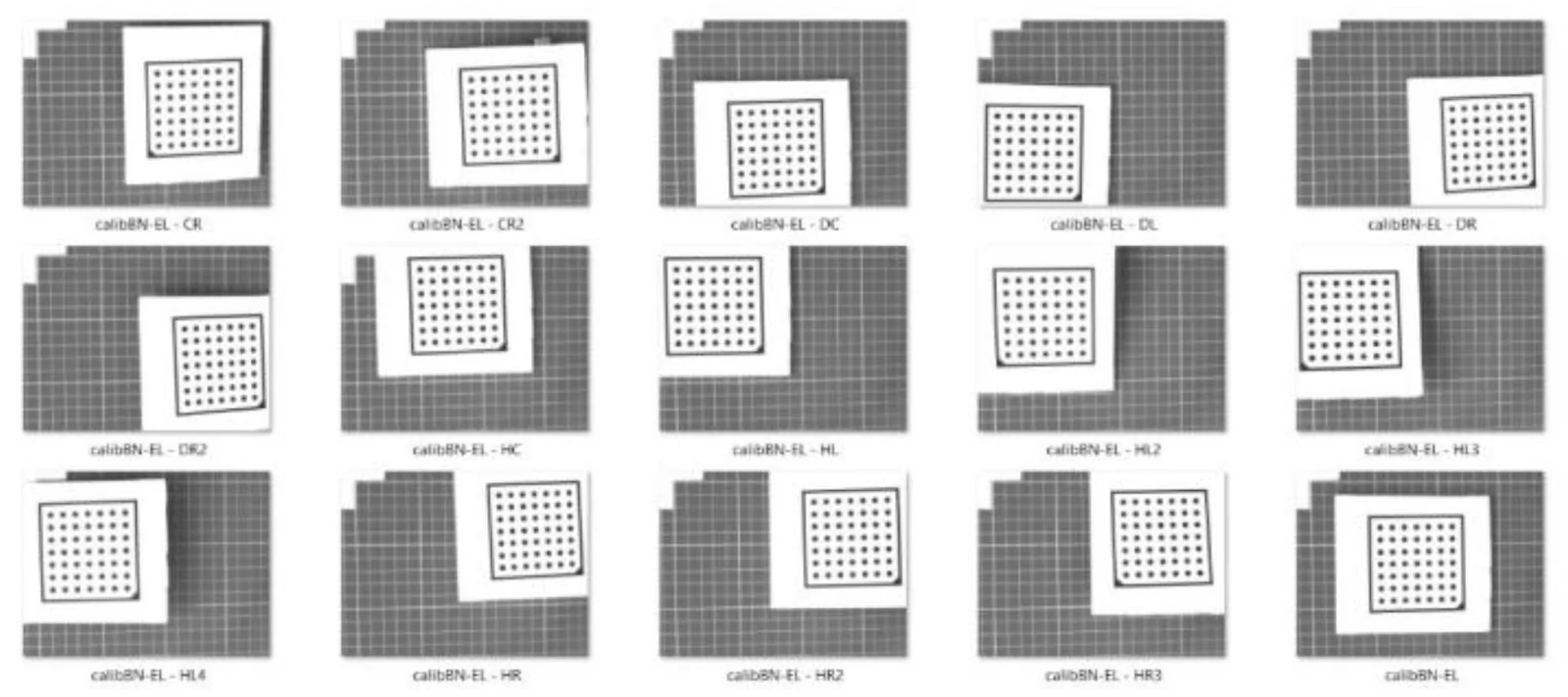
IDS NIR Camera calibration images.

**Fig. 6:**
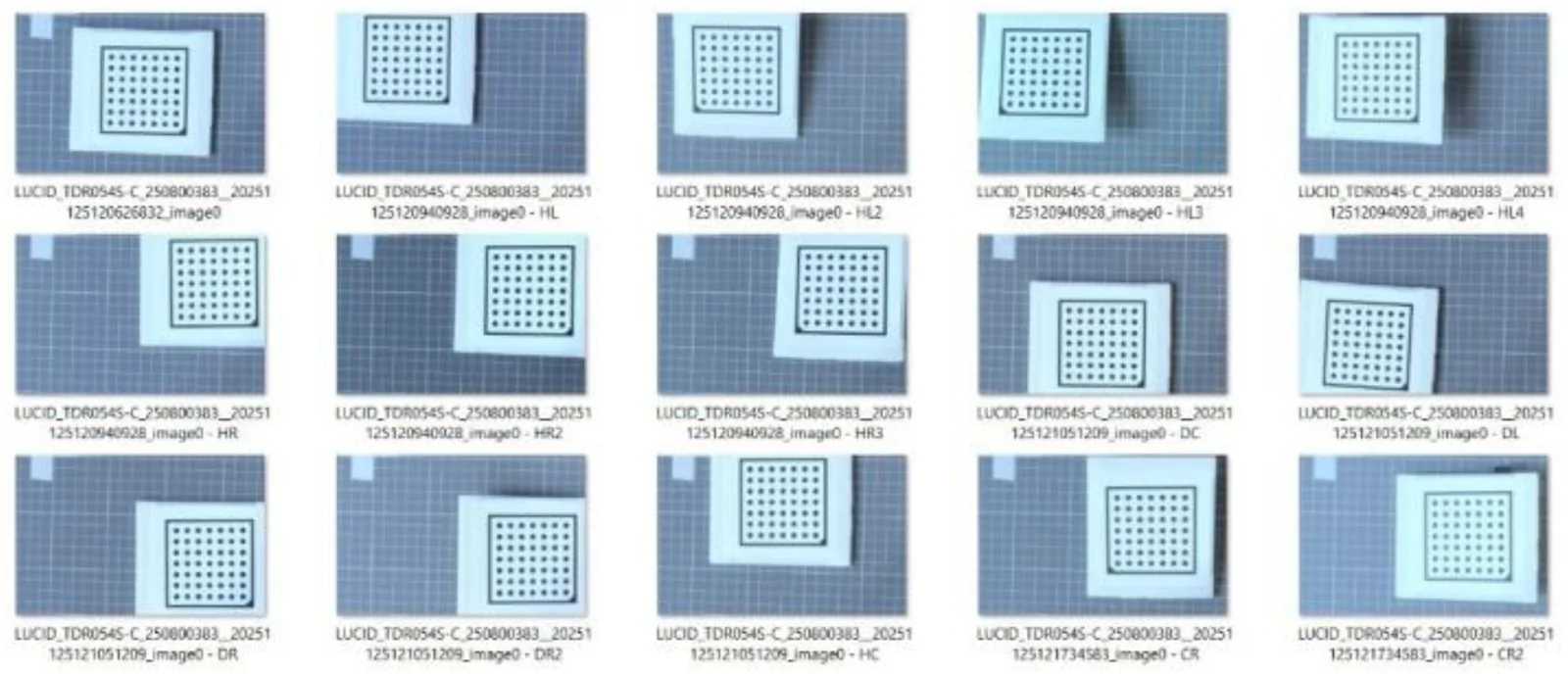
HDR LUCID Camera calibration images.

For both HDR and NIR camera mounted in the experimental setup, a set of calibration board images was acquired (Fig. 5, Fig. 6).

**Fig. 7:**
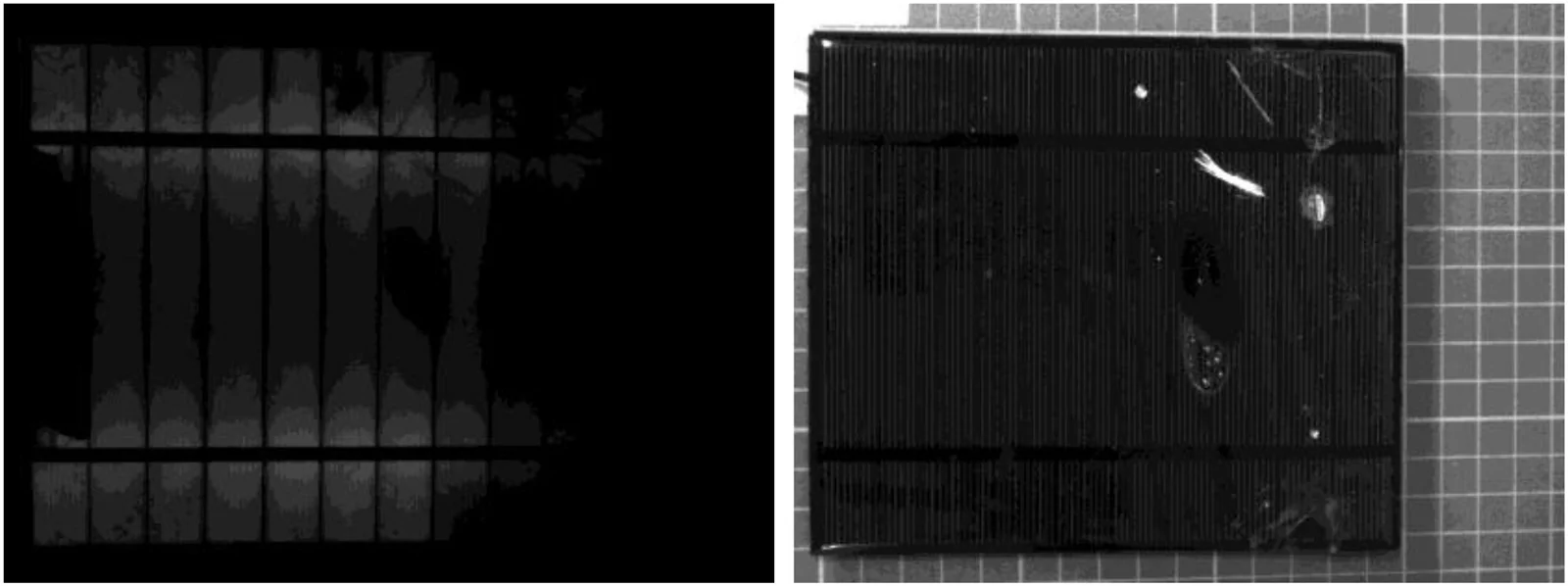
EL and NIR images: perfect pixel-to-pixel correspondence (specimen 01_D14).

**Fig. 8:**
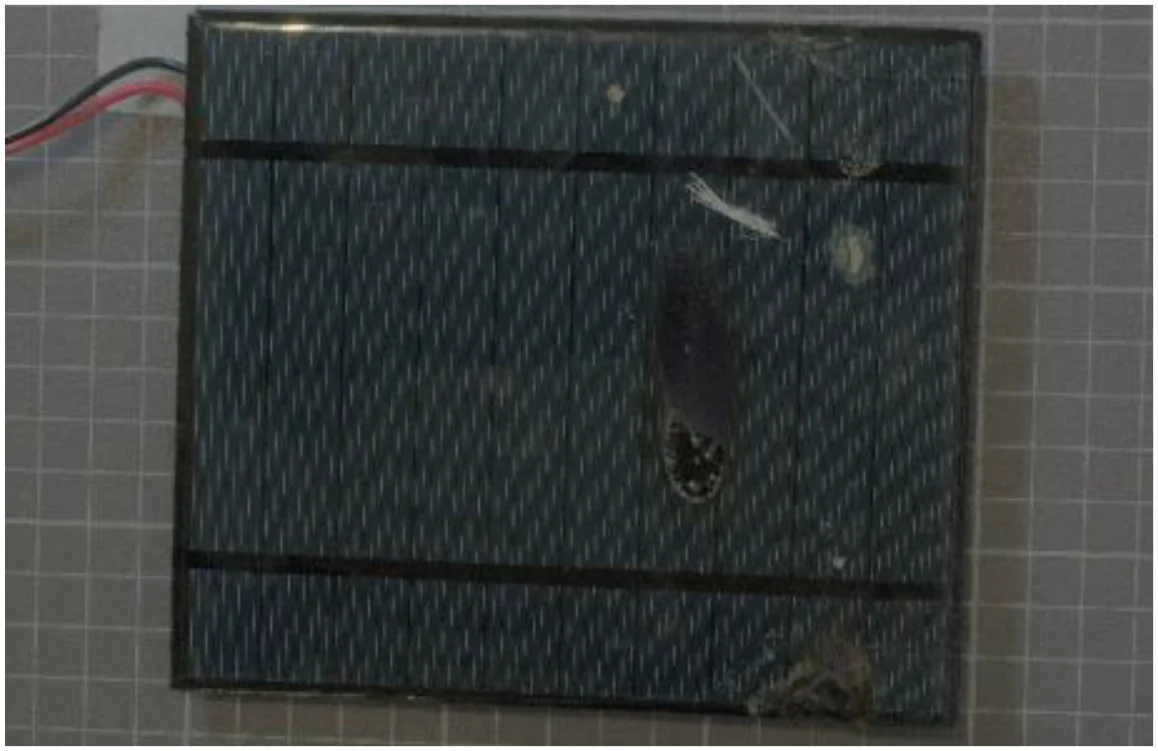
HDR picture (specimen 01_D14).

**Fig. 9:**
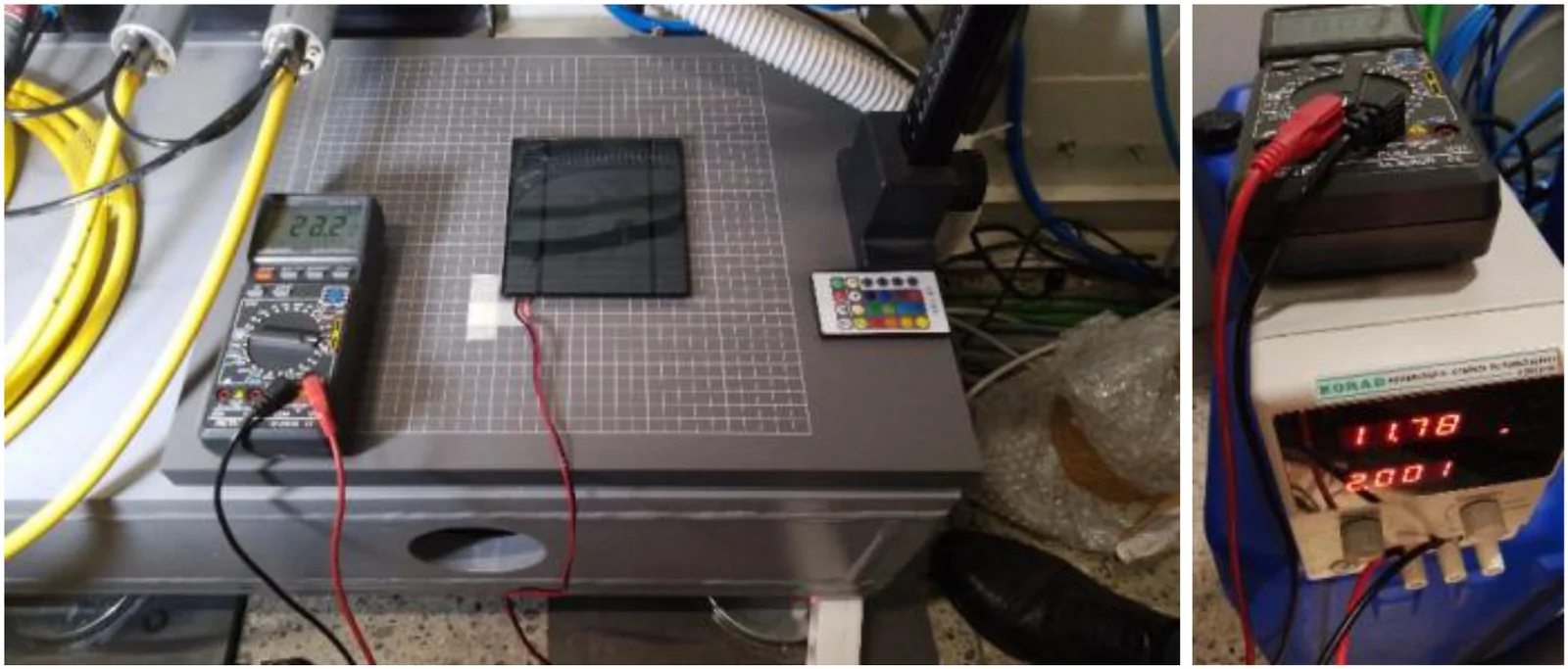
Electric performance measurement lab instruments.

Then, for both cameras, a calibration procedure was followed using MVTec HALCON: creating a calibration model; defining camera model (industrial camera, USB / GigE, global shutter, not telecentric lens); loading the previously generated calibration board descriptor .descr file; detecting the markers in each image; computing camera parameters; extracting and saving the intrinsic camera parameters (.cal file), including focal length, coordinates of the optical center, image resolution and distortion coefficients; extract and save (.dat file) the estimated camera extrinsic pose parameters, relative to the calibration board. After calibration, for each camera, a rectification map has been calculated to remove perspective distortion and register the images acquired by both cameras with an alignment uncertainty of a few pixels. Figs. 7, Fig. 8, Fig. 9

2. Specimen preparation

Each cell was incrementally damaged using lab tools (hammer; chisel; graver; screwdriver; cutter; soldering iron; lighter; pliers; wire cutters; bench vice; electrician's pliers; scraper) as described in the .CSV files. Some damage conditions were intentionally severe and not necessarily representative of typical field failures; these cases were included to increase defect variability within the dataset and support data-driven model development. The objective of the dataset is not to reproduce the complete temporal evolution of photovoltaic degradation processes, but to provide aligned multimodal observations of cells exhibiting different damage conditions for diagnostic, correspondence-learning, and generative modelling studies. Some samples may appear visually similar in the visible-spectrum modalities despite being associated with different degradation conditions. The dataset was designed to preserve these cases, as they provide examples where optical appearance alone is insufficient and complementary information from electroluminescence imaging and electrical measurements is required.

3. Image acquisition and electrical performance measurements

Data were acquired from a limited number of PV cells of a single manufacturer/model, as the experimental design prioritizes high-quality, pixel-level aligned multimodal acquisitions under controlled degradation conditions rather than population-scale diversity.

After damaging the PV cell, at each incremental damage, three images were acquired:

A. HDR
  During HDR image acquisition with ArenaView software, room lights were turned on as well as the halogen lamp; output V and A were recorded.
B. Monochrome NIR
  During NIR image acquisition with IDSpeak software, room lights were turned on; no power input was applied; no electric parameter was recorded.
C. EL

The electroluminescence image acquisition with IDSpeak software was driven by connecting each cell to a regulated power source in a darkened room and imposing a fixed voltage of 6.8 V, allowing current to adjust naturally and produce stable light emission for imaging. The electroluminescence emission was imaged using the NIR camera.

During EL, the room lights were turned off; the power source was turned on; input voltage and current were recorded for reference.

Then, for each cell’s damage condition corresponding to a 3-image set (HDR, NIR and EL), electric performances were recorded.

## 5 Image Post-Processing

*Post-processing pipeline overview*

For each 3-image set describing a single damage condition, image post-processing (Table 14) was applied using a MVTec HALCON procedure with the following steps:

A. Camera calibration loading
B. Calibration board detection
C. Measurement plane definition
D. World-plane rectification maps generation
E. Image rectification
F. Cell segmentation
G. Bounding box estimation
H. Consistent cropping across image types

**Table 14:** Summary table of calibration & rectification.

Camera/Image	Calibration Used	Rectification Map	Notes
EL	CameraParameters-EL.cal	EL_RectificationMap	Independent calibration
BN	CameraParameters-EL.cal	EL_RectificationMap	Shared map with EL because the same camera is used
HDR	CameraParameters-HDR.cal	HDR_RectificationMap	Independent calibration

MVTec HALCON function used and file names are listed in the following section.

*Post-processing pipeline details*

A. Camera Calibration loading

• Calibration A (EL camera): intrinsic parameters loaded from CameraParameters-EL.cal, with corresponding pose and calibration image. This calibration is used to generate the rectification map for both EL and NIR images.
  ○ Load the calibration file: read_cam_par('CameraParameters-EL.cal',CameraParameters)
  ○ Pose loaded from: CameraPose-EL.dat
  ○ Calibration image: calibBN-EL.png
  ○ Generates rectification map: EL_RectificationMap
• Calibration B (HDR camera): intrinsic parameters loaded from CameraParameters-HDR.cal, used to generate a separate rectification map.
  ○ Load the calibration file: read_cam_par('CameraParameters-HDR.cal',CameraParametersHDR)
  ○ Pose loaded from: CameraPose-HDR.dat
  ○ Calibration image: LUCID_TDR054S-C.jpg
  ○ Generates **rectification map**: HDR_RectificationMap

Note: NIR camera images use the same EL rectification map**.** This assumes perfect alignment and identical optics between EL and NIR, consistent with the use of the same camera, ensuring pixel-wise correspondence.

B. Calibration Board Detection

A planar calibration target is detected using a predefined descriptor file. The procedure includes the following steps:

• Load the .descr file describing the calibration board (caltab.descr)
• Create a calibration model (create_calib_data)
• Detect calibration board in reference images (find_calib_object)
• Extract calibration marks and compute pose (get_calib_data_observ_points)

Note: To account for the physical thickness of the calibration plate (2 mm), the pose is corrected by translating the origin along the optical axis, ensuring the measurement plane aligns with the real surface. [set_origin_pose(CameraPose, 0.0,0.0,−0.002,CameraPose)]

C. Measurement Plane Definition

The world coordinate system is defined such that:

• The origin is shifted to approximately center the calibration plate [set_origin_pose (CameraPose, …, TmpCtrl_RectificationPose)]
• The measurement plane corresponds to the actual object surface

Note: a user-defined metric width specifies the field of view in world coordinates.

D. World-Plane Rectification Maps Generation

World-plane rectification maps are generated for each calibrated camera. These maps enable transformation from image coordinates to metric world coordinates, removing perspective and lens distortions.

• EL/NIR: [gen_image_to_world_plane_map(EL_RectificationMap, CameraParameters, TmpCtrl_RectificationPose, …)]
• HDR: [gen_image_to_world_plane_map(HDR_RectificationMap, CameraParametersHDR, TmpCtrl_RectificationPose, …)]
• Note: these maps convert image coordinates to metric, undistorted world coordinates**.**

E. Image Rectification

To ensure geometrically consistent, distortion-free representations, all images are rectified using the corresponding precomputed maps:

• EL and NIR images use the same rectification map
  ○ EL camera: [map_image(Image_EL, EL_RectificationMap, EL_RectifiedImage)]
  ○ NIR camera: [map_image(Image_BN, EL_RectificationMap, BN_RectifiedImage)]
• HDR images use an independent rectification map
  ○ HDR camera: [map_image(Image_HDR, HDR_RectificationMap, HDR_RectifiedImage)]

Note: NIR (BW) camera is not independently calibrated; this is admissible only because the same camera is used

F. Cell segmentation

The panel is segmented from the rectified EL image using:

• Threshold on EL rectified image: [threshold(EL_RectifiedImage, Regions, 4, 255)]
• Connected component analysis [connection(Regions,ConnectedRegions)]
• Shape-based filtering (area and width constraints) [select_shape(SelectedRegions,SelectedRegions,'width','and',100,3300)]
• Merge regions to form the full cell mask [union1(SelectedRegions, RegionUnion)]
• The resulting regions are merged to obtain a single mask corresponding to the cell.

G. Bounding box estimation

A minimum-area oriented bounding rectangle is computed around the segmented panel. This provides:

○ Center position
○ Orientation angle
○ Principal dimensions

Bounding boxes are obtained with the following steps:

• Compute smallest rectangle around the panel (smallest_rectangle2)
• Extract rectangle parameters: center (Row, Column), orientation (Phi), dimensions (Length1, Length2) [smallest_rectangle2(RegionUnion, Row, Column, Phi, Length1, Length2)]

H. Consistent cropping across image types

All modalities (EL, NIR, HDR) are cropped using the same geometrical reference derived from the EL segmentation:

• Crop EL, NIR, and HDR images using the same bounding rectangle with small offsets
• Rotate/correct orientation according to Phi

This ensures spatial alignment across modalities and produces images suitable for downstream analysis.

Note: a minor empirical adjustment was applied during post-processing to compensate for residual misalignment in the estimated extrinsic parameters.

○ crop_rectangle2(EL_RectifiedImage, EL_RectifiedImage_crop, Row-248, Column-10, Phi+0.00425, Length1+140, Length2+120, 'true', 'constant')
○ crop_rectangle2(HDR_RectifiedImage, HDR_RectifiedImage_crop, Row-243, Column-210, Phi+0.013, Length1+140, Length2+120, 'true', 'constant')
○ crop_rectangle2(BN_RectifiedImage, BN_RectifiedImage_crop, Row-248, Column-10, Phi+0.00425, Length1+140, Length2+120, 'true', 'constant')

Due to the use of the same camera for EL and NIR, for these images there is a perfect pixel-wise correspondence, reflected in the same empirical adjustment.

## 6 Image Labelling

After the acquisition of the 3-image set for each damage condition, a visual inspection has been performed and a detailed textual description of the images, of the damage condition and of the electric performances was recorded.

## Limitations

• Limited dataset size (84 damage conditions)
• Limited number of PV cells and single cell technology, which restricts statistical generalization and large-scale representativeness, as the dataset is designed for controlled multimodal correspondence learning rather than population-level degradation analysis.
• Electroluminescence images acquired by a camera equipped with a silicon CMOS sensor with lower quantum efficiency in the near infrared range than cameras with InGaAs sensors
• Low irradiance of the test light source
• Limited test instruments' accuracy
• Variable temperature conditions (both of cells and environment)
• Limited tolerance of image registration algorithm based on camera calibration to off-plane deformations due to thermal and mechanical stresses
• Cell-deformation and cell-positioning dependent manual fine-tuning of automated rectification and alignment procedure
• Potential observer bias arising from subjective annotation procedures lacking inter-rater agreement assessment
• Irradiance value estimated via photometric-to-radiometric conversion rather than direct calibrated radiometric measurements, introducing uncertainty in the absolute irradiance level.

## Ethics Statement

The authors have read and follow the ethical requirements for publication in Data in Brief and confirm that the current work does not involve human subjects, animal experiments, or any data collected from social media platforms.

## CRediT Author Statement

**Marcello Polenghi**: Conceptualization, Methodology, Writing - Original draft, Software, Validation, Data acquisition and Investigation, Formal analysis, Resources, Data Curation, Visualization, Writing - Review & Editing; **Vittorio Sala**: Methodology, Software, Validation, Data acquisition and Investigation, Supervision, Review & Editing; **Emanuele Ogliari**: Conceptualization, Methodology, Resources, Supervision, Review & Editing; **Roberto Caldelli**: Supervision, Review & Editing. **Claudio Loconsole**: Supervision, Review & Editing**; Anna Valente:** Resources, Review & Editing; **Ambra Vandone** Resources, Review & Editing

## Data Availability

Mendeley DataPV_EL_HDR_BW_Test_DB (Original data).
